# Validity, reliability and minimum detectable change of COSMED K5 portable gas exchange system in breath-by-breath mode

**DOI:** 10.1371/journal.pone.0209925

**Published:** 2018-12-31

**Authors:** Laura Guidetti, Marco Meucci, Francesco Bolletta, Gian Pietro Emerenziani, Maria Chiara Gallotta, Carlo Baldari

**Affiliations:** 1 Department of Movement, Human and Health Sciences, University of Rome “Foro Italico”, Rome, Italy; 2 Vascular Biology and Autonomic Studies Laboratory, Appalachian State University, Boone, North Carolina, United States of America; 3 Department of Experimental and Clinical Medicine, University of Magna Græcia of Catanzaro, Catanzaro, Italy; 4 eCampus University, Novedrate (Como), Italy; Sao Paulo State University - UNESP, BRAZIL

## Abstract

**Purpose:**

This study aimed to examine the validity, reliability and minimum detectable change (MDC) of the Cosmed K5 in breath by breath (BxB) mode, against VacuMed metabolic simulator. Intra and inter-units reliability was also assessed.

**Methods:**

Fourteen metabolic rates (from 0.9 to 4 L.min-1) were reproduced by a VacuMed system and pulmonary ventilation (VE), oxygen consumption (VO_2_) and carbon dioxide production (VCO_2_) were measured by two different K5 units. Validity was assessed by ordinary least products (OLP) regression analysis, Bland-Altman plots, intraclass correlation coefficients (ICC), mean percentage differences, technical errors (TE) and MDC for VE, VO_2_, and VCO_2_. Intra- and inter-K5 reliability was evaluated by absolute percentage differences between measurements (MAPE), ICCs, TE, and MDC.

**Results:**

Validity analysis from OLP regression data and Bland- Altman plots indicated high agreement between K5 and simulator. ICC values were excellent for all variables (>0.99). Mean percentage differences in VE (-0.50%, p = 0.11), VO_2_ (-0.04%, p = 0.80), and VCO_2_ (-1.03%, p = 0.09) showed no significant bias. The technical error (TE) ranged from 0.73% to 1.34% (VE and VCO_2_ respectively). MDC were lower than 4% (VE = 2.0%, VO_2_ = 3.8%, VCO_2_ = 3.7%). The intra and inter K5 reliability assessment reveled excellent ICCs (>0.99), MAPE <2% (no significant differences between trials), TE < or around 1%, MDC <or around 3%.

**Conclusions:**

K5 in BxB mode is a valid and reliable system for metabolic measurements. This is the first study assessing the MDC accounting only for technical variability reporting intra- and inter-units MDCs <3.3%.

## Introduction

The use of automated metabolic systems to measure oxygen consumption (VO_2_) and carbon dioxide production (VCO_2_) has become an essential tool for the analysis of physical performance and clinical diagnoses. Over the last three decades, the development of technology has facilitated the transition from laboratory to field measurements by introducing a variety of portable systems able to measure the energy cost of outdoor activities [1, 2].

COSMED recently launched a new portable metabolic system, (Cosmed K5) culminating in significant hardware, firmware and software improvements from the previous model. The K5 is a single unit device (174×64×114 mm dimensions and ~900 g weight) combining breath-by-breath (BxB) technology from the COSMED K4b2 and the dynamic mixing chamber system used in the COSMED Fitmate series. This option, called ‘IntelliMET’ (Intelligent Dual Metabolic Sampling Technology), allows users to select either the dynamic mixing chamber or the BxB sampling modality to measure either steady-state metabolic rates or oxygen kinetics during transients. This technology is supported by a series of significant hardware and firmware/software updates that aim to improve the reliability of its’ measures: 1) a dynamic mixing chamber that uses a constant flow pump; 2) a 4th generation opto-electronic reader and high performance turbine flowmeter with 0.08–16 L/s flow range; 3) an external scrubber to obtain real zero carbon dioxide and allow for more accurate gas calibration; 4) an external ambient temperature sensor for the calculation of the inspiratory BTPS factor and a capacitive ambient humidity and piezo-resistive pressure sensors inside the K5 unit for the calculation of the expiratory BTPS and STPD factors. Additional functions have been included to improve flexibility and durability of the product such as a 3.5″ TFT back-lit LCD touch-screen; a 4h Li-ion “smart battery”, an integrated 10 Hz GPS receiver for navigation/motion, integrated ANT+ technology for optional wireless sensors, a weatherproof case (IP54 standard), a standard or long-range Bluetooth 2.1 and an SDHC card for additional data storage [2].

The aim of the present study is to evaluate the validity and reliability of the COSMED K5 with BxB measurements, testing two different portable units over a wide range of metabolic rates. Douglas bag methods have been used to determine accuracy and precision of cardiopulmonary exercise testing (CPET) equipment, however previous studies clearly state limits to this method. Inherent biological variability disproportionately contributes to the overall error with Douglas bags whereas only a small part of the variability is caused by the measurement itself [3, 4]. On the other hand, metabolic simulators [5] are able to remove the biological variability and isolate measurement errors by systematically reproducing the human breath [6, 7]. Preliminary data produced in our laboratory, published as congress abstracts, suggest that the system is adequately reliable and valid when compared against a criterion VacuMed metabolic simulator [8, 9], however, a systematic validation study is necessary.

## Methods

The study was conducted within the Department of Health Sciences at the University of Rome “Foro Italico”. COSMED and VacuMed were not involved in designing the study, data collection, analysis, interpretation or preparation of the manuscript.

### COSMED K5

Two COSMED K5 units, s/n 2015060002 (K5_02) and 2015060018 (K5_18), were used in this study. The K5 system uses a galvanic fuel cell and a non-dispersive infrared sensor for the analysis of oxygen (O_2_) and carbon dioxide (CO_2_) in the inhaled and exhaled air and an opto-electronic reader with a high performance turbine flowmeter to measure flow rate. After 30 minutes of warm up; flowmeter, gas, scrubber and delay time calibrations were performed following manufacturer’s recommendations. The two-point gas calibration was completed sampling the ambient air and the gas from a certified tank containing 16% O_2_, 5% CO_2_ and standard atmospheric Nitrogen. A 0% CO_2_ sampling was performed using a CO_2_ scrubber to obtain an accurate 0% CO_2_ reading and adjust for the CO_2_ and O_2_ values in the atmospheric air. Flowmeter calibration was performed connecting the turbine to a calibrated Hans Rudolph 3-liter syringe and completing six full strokes at a respiratory frequency of 20–25 b/min. Delay time calibration was performed with the flowmeter and the sampling line connected to the face mask and by executing six breaths at a given rhythm while breathing in the facemask.

### VacuMed automated system

A commercially available metabolic simulator, the VacuMed automated system model #17056 (VacuMed, USA), was used in this study. This system uses a motor-drive syringe able to vary tidal volume and respiratory frequency to reproduce different ventilations (VE), and a gas tank containing air with the 79% N_2_ and 21% CO_2_ that is used to reproduce the VO_2_ and VCO_2_ in the exhaled air at different metabolic rates by diluting the gas tank with room air inside the piston pump [6]. Therefore, VO_2_ and VCO_2_ are proportional to the gas flow of calibration gas from the tank. Simulated volumes have been automatically corrected by the VacuMed software that compensated for temperature, barometric pressure and humidity measured in room air since VO_2_ and VCO_2_ are expressed in STPD while the simulator system utilizes known mixtures of a dry tank gas with a partially humidified room air [10]. The manufacturer certifies a system accuracy of 1% for simulated VO_2_ and VCO_2_ and a 0.25% for tidal volume.

### Study design

**Fig 1:**
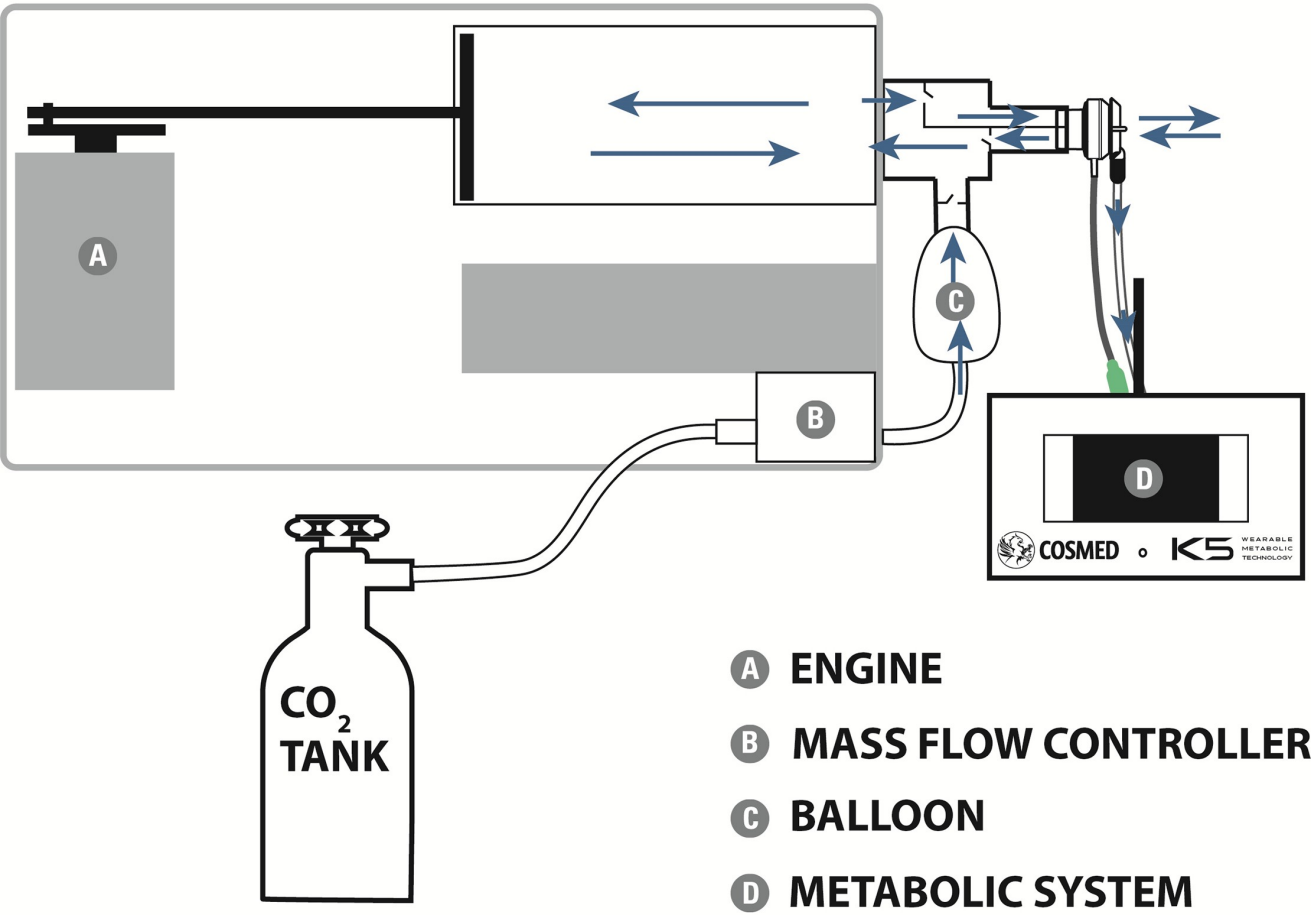
Schematic of the COSMED K5 and VacuMed systems.

The flowmeter and sampling line of each COSMED K5 system were connected directly to the outlet of the VacuMed automated system. Fig 1 shows a schematic of the system.

The K5_02 and K5_18 were tested separately and on different days under similar atmospheric conditions. The COSMED K5 units measured the volume and the concentrations of O_2_ and CO_2_ in each breath exhaled by the VacuMed system. Fourteen metabolic rates (from 0.9 to 4 L^.^min^-1^) were simulated by the VacuMed system and measured by the two K5 units. All tested metabolic rates are reported in Table 1.

**Table 1:** Protocol used to simulate 14 different metabolic rates (step) using VacuMed simulator.

	Simulator settings			Simulated values		
Step	Pump Stroke Volume (L)	Pump Stroke Rate (rev^.^min^-1^)	Mass Flow of Cal Gas (L^.^min^-1^)	VE (L^.^min^-1^)	VO_2_ (mL^.^min^-1^)	VCO_2_ (mL^.^min^-1^)
1	1.5	15	4.5	25	941	952
2	1.5	25	6.0	42	1252	1268
3	1.5	30	8.0	49	1670	1692
4	1.5	40	9.0	65	1878	1904
5	2	20	7.0	44	1459	1478
6	2	35	9.5	77	1980	2009
7	2	40	11.0	88	2293	2325
8	2	55	14.0	120	2922	2960
9	2.5	35	12.0	97	2504	2536
10	2.5	40	13.0	111	2716	2729
11	2.5	55	18.0	152	3760	3778
12	3	40	15.5	136	3238	3253
13	3	45	17.5	149	3658	3673
14	3	50	19.0	166	3969	3988

“Cal Gas”; calibration gas mixture (21% CO_2_ and 79% N_2_, as detailed in VacuMed manual)

Atmospheric pressure, ambient temperature, and relative humidity were measured by the K5 units before each test. Expired gases were sampled at the turbine through a semipermeable Nafion sampling line (0.75 m in length), and analyzed into the COSMED K5 portable units through an electro-galvanic fuel O_2_ cell and an infrared CO_2_ analyzer. All data were transmitted by Bluetooth from the portable unit to a personal computer and controlled in real time. Data from each metabolic rate were measured BxB for 70 s and the values were entered into a spreadsheet for later analysis. Raw data were reduced by removing the first 10 s of measurement to eliminate data related to the wash-out of the gas-filled dead space of the simulator, and performing a 60 s average of the remaining breaths. The accuracy and reliability of the K5 units were assessed for the main ventilatory and gas exchange variables: VE (L^.^min^-1^), VO_2_ (mL^.^min^-1^), and VCO_2_ (mL^.^min^-1^).

### Statistical analyses

#### Validity

Agreement between the COSMED K5 and the VacuMed systems were assessed for VE, VO_2_, and VCO_2_ parameters by ordinary least products (OLP) regression analysis, which account for measurement error in both devices [11]. Regression parameters (slope and intercept), coefficients of determination (R^2^), and 95% confidence intervals (95% CI) were calculated for the OLP regression equations to determine fixed and proportional biases. The 95% confidence intervals containing the value 1 for the slope and the 0 for the intercept allows rejecting the hypothesis of proportional and fixed differences respectively. Bland-Altman plots [12] were constructed to determine the 95% limits of agreement (LoA) between the COSMED K5 and the VacuMed systems. Intraclass correlation coefficients (ICC) were used as parameters for criterion validity of the Cosmed K5 compared to the VacuMed simulator. A single measure, two-way random model, type absolute intra-class correlation coefficient was used to calculate ICCs. The strength of criterion reliability for ICC was classified in accordance with Hopkins (2000) [13].

Lastly, accuracy was quantified as the percentage differences (error) between the COSMED K5 s and VacuMed simulator [100*(COSMED K5-VacuMed)/VacuMed] and reported as mean and range values. COSMED K5 validity was also assessed by comparing the measured VE, VO_2_, VCO_2_ values vs simulated values with a paired samples t-test. Measurement error was expressed in “typical percentage error” (TE) and “minimum detectable change” (MDC). Typical error was calculated by dividing the standard deviation of the difference score by √2. This typical percentage error is a coefficient of variation and is considered highly reliable if less than 5% [13]. MDC values [also referred to as the “smallest detectable difference (SMD)], which reflects the magnitude of change necessary to provide confidence that the change was not resultant of random variation or measurement error, were calculated as 1.96*√2*TE.

#### Reliability

To verify intra- and inter-K5 reliability the ICCs were determined on the 14 simulated metabolic rates measured twice by the same system or by two different K5 systems, respectively. A single measure, two-way mixed model, type absolute intra-class correlation coefficient was used to calculate ICCs [13, 14]. Intra and inter COSMED K5 system differences were quantified as the absolute percentage differences between measurements of the same K5 or between two different K5 systems, respectively. Due to the lack of a reference system, percentage difference was calculated as absolute percentage difference divided by the average intra or inter-system values and multiplied by 100. COSMED K5 BxB reliability was also assessed by comparing both intra and inter-system measures of VE, VO_2_, VCO_2_ with a paired samples t-test. Measurement error of intra- and inter-K5 systems was expressed in TE and MDC and calculated as reported in the validity section.

Statistical analyses were performed using the SPSS software package version 24.0 (SPSS Inc., Chicago, IL, USA), with a significance level set at p < 0.05.

## Results

### Validity

Table 2 details R^2^, parameters of the OLP regression equation (slope and intercept) and the mean percentage of the difference between the values generated by the VacuMed simulator and measured by the K5. The agreement between values generated by the VacuMed simulator and measured by the K5 for the main gas exchange variables is presented in Table 2.

**Table 2:** Agreement between values generated by the VacuMed simulator and values measured by the K5 as assessed by OLP regression analysis.

	R^2^	Slope (95% CI)	Intercept (95% CI)	mean % diff (min to max)	*p*	ICC (95% CI)	**TE**	**MDC**
VE (L^.^min^-1^)	0.9995	0.994 (0.980 to 1.008)	0.063 (-1.283 to 1.391)	-0.50 (-2.79 to 0.66)	0.11	1.000 (0.999 to 1.000)	0.73	2.01
VO_2_ (mL^.^min^-1^)	0.9969	0.984 (0.950 to 1.018)	36.5 (-48.6 to 118.7)	-0.04 (-2.55 to 3.52)	0.80	0.999 (0.998 to 1.000)	1.37	3.79
VCO_2_ (mL^.^min^-1^)	0.9973	0.983 (0.952 to 1.016)	15.9 (-64.3 to 93.4)	-1.03 (-3.82 to 2.13)	0.09	0.999 (0.997 to 1.000)	1.34	3.71

Determinant coefficient (R^2^), slope and intercept of the regression equations, as well as mean percentage difference (mean % diff), p values, intra-class correlation coefficient (ICC), typical percentage error (TE), and minimum detectable change (MDC) are reported.

**Fig 2:**
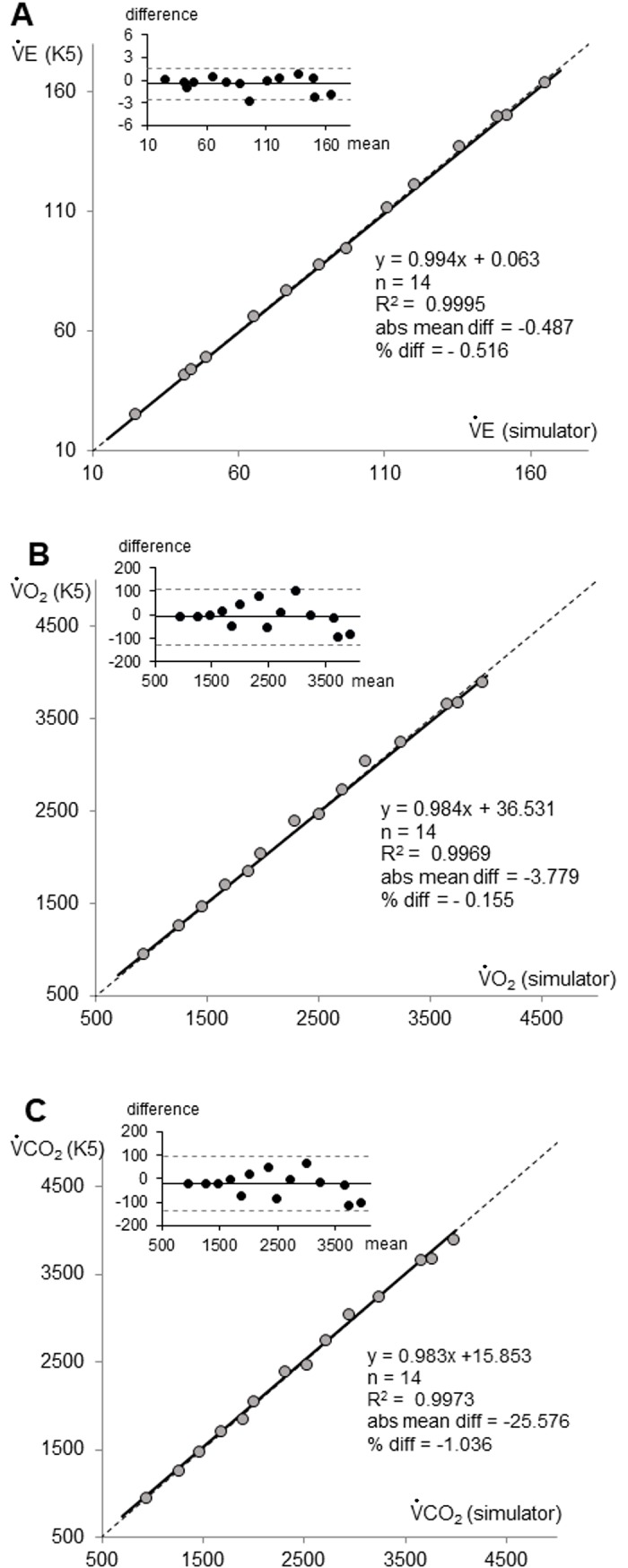
Regression and difference plots of pulmonary ventilation (VE), oxygen uptake (VO_2_) and carbon dioxide production (VCO_2_) measured by K5 and generated by the VacuMed simulator.

VacuMed and K5 values demonstrated high correlation (R^2^ > 0.99; ranging from 0.9969 to 0.9995) in VE, VO_2_ and VCO_2_ variables. OLP regression analysis reported slope and intercept values that always include the 1 and the 0, respectively. ICC values were excellent for all variables (>0.99). The OLP regression analysis and the Bland-Altman plots of the averaged VE, VO_2_, and VCO_2_ values obtained during the 14 simulated metabolic rates between the Simulator (VacuMed) and K5 (BxB modality) are graphically shown in Fig 2A, 2B and 2C respectively.

For each one of the 3 main graphs is the OLP regression plot (the outside panel), with the linear regression (solid line), the identity (dashed line) and the equation with the Pearson’s determinant coefficient (R^2^), and the Bland-Altman plot (upper-left panel) with the mean difference (solid lines) and the 95% CI (dashed lines).

Mean percentage differences in VE (-0.50%, p = 0.11), VO_2_ (-0.04%, p = 0.80), and VCO_2_ (-1.03%, p = 0.09) showed no significant bias (Table 2). The typical percentage error (TE) ranged from 0.73% to 1.34% (VE and VCO_2_ respectively). MDC was lower than 4% (VE = 2.0%, VO_2_ = 3.8%, VCO_2_ = 3.7%).

### Reliability

ICC was excellent (>0.99) in all conditions (Table 3). For all variables, the mean absolute percentage error (MAPE), calculated intra- and inter- K5 system were around 1% with the 95% CI values below 3%, and no significant differences were found between trials. The typical percentage error was below or around 1%. The MDC values were similar or slightly lower in the intra- (VE = 2.0%, VO_2_ = 2.3%, VCO_2_ = 2.6%) when compared to the inter-K5 system reliability (VE = 2.0%, VO_2_ = 3.2%, VCO_2_ = 3.3%).

**Table 3:** Intra- and inter-system K5 reliability.

	Intra-K5 system reliability					Inter-K5 systems reliability				
	MAPE (95% CI)	*p*	ICC (95% CI)	TE	MDC	MAPE (95% CI)	*p*	ICC (95% CI)	**TE**	**MDC**
VE (L^.^min^-1^)	0.66 (0.08 to 1.23)	0.90	1000 (1.000 to 1.000)	0.71	1.96	0.99 (0.41 to 1.58)	0.73	1000 (0.999 to 1.000)	0.72	1.99
VO2 (mL^.^min^-1^)	1.11 (0.44 to 1.78)	0.41	0.999(0.997to 1.000)	0.82	2.28	1.36 (0.91 to 1.81)	0.65	0.999 (0.997 to 1.000)	1.15	3.19
VCO2 (mL^.^min^-1^)	1.16 (0.86 to 2.37)	0.98	0.999 (0.997to 1.000)	0.93	2.57	1.85 (1.35 to 2.35)	0.68	0.998 (0.995 to 0.999)	1.18	3.28

Mean absolute percentage difference (MAPE), intra-class correlation coefficient (ICC), p values, typical percentage error (TE), and minimum detectable change (MDC) are reported.

## Discussion

The first aim of this study was to test the accuracy of the COSMED K5 portable metabolic measurement system using the BxB setting against the criterion VacuMed simulator. The second aim assessed the intra- and inter-K5 system reliability. To our knowledge, this is the first study assessing accuracy and reliability of the K5 portable metabolic system in comparison with a gas exchange simulator. However, metabolic simulators have been widely used in the past to assess the validity of different metabolic systems [15–17].

### Validity

The results indicate high agreement between the K5 measurements and the simulated values over a wide range of simulated exercise intensities (VO_2_ up to 4 L^.^min^-1^). The OLP regression equations indicated that neither fixed or proportional biases were present. All ICCs and 95%CI values showed excellent agreement > 0.99. The OLP regression analysis revealed no proportional or fixed differences between measured (K5) and simulated (VacuMed) in all variables (VE, VO_2_, VCO_2_). Measurement differences were within ±4% and in agreement with the suggested range of differences for VO_2_ [18]. Mean differences were less than -1% for VE (-0.5%) and VO_2_ (-0.04%) and -1% for VCO_2_, with no statistical significance between measured and simulated values. Previous studies reported higher differences when comparing simulated and measured values when the K4b2 (VE 4.2%, VO_2_ 3.6%, VCO_2_−2.2%) (16), the Quark CPET or an automated on-line system (4–12% range) were used [15, 17]. Moreover, our results reported a typical percentage error of 0.7% for VE and 1.4% and 1.3% for VO_2_ and VCO_2_, respectively, which is lower than the < 3% VO_2_ and <5% VE recommended by Hodges at al. (2005) [19]. Even in the case when it could be extremely likely that these two reference percentages were erroneously attributed, (VE < 3% and VO_2_ < 5% since VO_2_ derives from a calculation that includes VE and FO_2;_ [20, 21], these results will still be considered below the acceptable percentage of error. We hypothesized the 1% error we observed in VE may depend on the small temperature difference between simulated inspired and expired air as these are close to room temperature, contrasted by *in vivo* measurements where the expired temperature is set by default to 34°C. This may suggest that a real temperature measurement of expired air could positively influence the accuracy of VE values during *in vivo* measurements, even though it has been noted that a 1.0°C difference in the estimated expiratory temperature from the actual temperature would result in only a 0.6% error in VE having only a minor effect on the calculation of VO_2_ [22].

To our knowledge, previous studies used the MDC to evaluate the reliability of a test protocol and a metabolic system [23, 24] but it has never been used versus a criterion system (simulator) to quantify the minimum change attributing the difference to the measurement error and not to the result of random variation. If the difference between a single measurement and a criterion is smaller than the smallest detectable change, it is likely due to measurement error while any difference larger than the MDC should be considered as real difference. In our study, the MDC of the simulator for VE (2%) was lower than the accuracy limits reported by ATS/ERS guidelines for spirometry of 3.5% [21]. Moreover, most certification bodies tolerate a maximal error of 4% in VO_2_ [19, 25] and a difference <5% versus the reference method would be considered as acceptable [1]. In our study the MDC in VO_2_ (3.8%) and VCO_2_ (3.7%) were lower than the reference values from literature for VO_2_ and reflect the low MDC value in VE (2%). Lastly, the low MDC values reported in VO_2_ and VCO_2_ reflect the low MDC in VE, since that the accuracy of VO_2_ and VCO_2_ in the BxB calculation is influenced by both VE and time delay errors [17].

### Reliability

K5 in breath-by-breath mode showed an excellent intra- and inter- device reliability in VO_2_ and VO_2_ with ICCs >0.99 and a MAPE <1.5%. In previous studies the ICCs test-retest reproducibility ranged between 0.90 to 0.97 for stationary metabolic carts [26, 27], and between 0.88 to 0.95 for portable systems such as K4b2 [28]. The highest ICCs were obtained for mixing chamber systems (0.98 and 0.98, VO_2_ and VCO_2_ respectively) and stationary breath by breath apparatus (0.97 and 0.96, O_2_ and VCO_2_, respectively) [29].

Studies that assessed test-retest variability in humans, obtained MAPE and coefficient of variation (CV) of 1.8 to 7.4% for VO_2_ and 4.1 to 7.7% for VCO_2_ including both technological errors and biological fluctuations [15, 26–28]. To our knowledge, the intra- and inter-unit technological variability was previously evaluated by connecting a metabolic system to a gas simulator [30] or attaching two devices to the exercising subject for simultaneous sampling [31]. These studies showed low intra-unit and inter-unit variability with relative percentage errors < 2% for VE, VO_2_ and VCO_2_ and TEM< 1.5%, and MAPE = 2.1% for VO_2_ and a CV = 1.5%, respectively. These results are similar or slightly higher than those obtained from our intra- and inter-device TEM (<1% and <1.2% for all VE, VO_2_ and VCO_2_), comparable to the 1% relative error generated from an automated calibration system [7] and considerably below the TEM reliability limit of 3% recommended by the Australian Sports Commission [32].

Previous reliability studies reported an MDC of 7–10% using human subjects which included both technical and biological variability [23, 33]. This is the first study assessing the MDC accounting only for technical variability. The inter-unit MDC of the K5, representing the smallest change detectable by the instrument beyond the variability of the technical measurement, was low (2 to 3.3%) and similar to the 2–2.6% intra-unit MDC for VE, VO_2_ and VCO_2_.

Limitations of this study are represented by the limited range of simulated metabolic rates and by the use of a gas exchange simulator. Some caution should be taken for metabolic rates lower and higher than those used in this study. Further, a physiological scope outside of the parameters we tested will affect the generalizability of these results. Moreover, despite the clear advantages of using a gas exchange simulator (e.g., exclusion of biological variability and reliable simulation of gas volumes in a wide range of measurements), limitations are represented by the production of ambient temperature and dry gases only mathematically corrected using the manufacturer’s software [10]. Despite these limitations, this study was able to assess validity and reliability of the COSMED K5 using BxB mode while only accounting for the variability caused by technical errors.

## Conclusions

The COSMED K5 in BxB mode is a valid system for the measurement of VE, VO_2_ and VCO_2_ for a wide range of metabolic rates as indicated by: a) the absence of systematic and proportional errors, b) the very high ICCs and excellent agreement c) a typical percentage error lower than 1.5%, d) the low MDC of 2–3.8% versus the reference system. The K5 was also found to be a reliable system as shown by the very high ICCs and the low intra and inter-device variability (TEM < 1.2%). Moreover, the low intra- and inter-device MDC (<2.6% and <3.3%, respectively) in repeated measurements is useful to discriminate measurement error from true change.

## Supporting information

S1 File Supporing information file. VCO2_sim = simulated carbon dioxide production; VCO2_K5_18_2 = carbon dioxide production measured by K5 unit 2015060018 as first measurement; VO2_sim = simulated oxygen uptake; VO2_K5_18_2 = uptake measured by K5 unit 2015060018 as first measurement; VE_sim = simulated pulmonary ventilation; VE_K5_18_2 = pulmonary ventilation measured by K5 unit 2015060018 as first measurement; VE_K5_18_1 = pulmonary ventilation measured by K5 unit 2015060018 as second measurement; VO2_K5_18_1 = oxygen uptake measured by K5 unit 2015060018 as second measurement; VCO2_K5_18_1 = carbon dioxide production measured by K5 unit 2015060018 as second measurement; VE_K5_02_1 = pulmonary ventilation measured by K5 unit 2015060002 as first measurement; VE_K5_02_2 = pulmonary ventilation measured by K5 unit 2015060002 as second measurement; VO2_K5_02_1 = oxygen uptake measured by K5 unit 2015060002 as first measurement; VO2_K5_02_2 = oxygen uptake measured by K5 unit 2015060002 as second measurement; VCO2_K5_02_1 = carbon dioxide production by K5 unit 2015060002 as first measurement; VO2_K5_02_2 = carbon dioxide production measured by K5 unit 2015060002 as second measurement.
(XLSX)
